# Prognostic factors in non-Hodgkin lymphomas

**DOI:** 10.1590/S1516-31802000000100003

**Published:** 2000-01-02

**Authors:** Karin Zattar Cecyn, José Salvador Rodrigues de Oliveira, Antônio Correia Alves, Maria Regina Regis Silva, José Kerbauy

**Keywords:** Non-Hodgkin lymphoma, Prognostic Factors, Survival, Linfoma não-Hodgkin, Fatores prognóstico, Sobrevida

## Abstract

**CONTEXT:**

In Hodgkin's disease, each clinical or pathologic stage can be related to the extent of the area involved and predicts the next anatomical region at risk for tumor dissemination.

**OBJECTIVE:**

To determine the best prognostic factors that could predict survival in non-Hodgkin lymphoma cases.

**DESIGN:**

A retrospective study.

**LOCATION:**

Department of Hematology and Transfusion Medicine, Universidade Federal de São Paulo - Escola Paulista de Medicina.

**PARTICIPANTS:**

142 patients with non-Hodgkin lymphoma diagnosed between February 1988 and March 1993.

**MAIN MEASUREMENTS:**

Histological subset, Sex, Age, Race, B symptoms, Performance status, Stage, Extranodal disease, Bulk disease, Mediastinal disease, CNS involvement, BM infiltration, Level of DHL, Immunophenotype.

**RESULTS:**

In the first study (113 patients), the following variables had a worse influence on survival: yellow race (P<0.1); ECOG II, III e IV (P<0.1) and extranodal disease (P<0.1) for high grade lymphomas; constitutional symptoms (P<0.1), ECOG II, III e IV (P<0.1) and involvement of CNS (P<0.1) for intermediate grade and the subtype lymphoplasmocytoid (P=0.0186) for low grade lymphomas. In the second survey (93 patients), when treatment was included, the variables related to NHL survival were: CNS involvement (P<0.1) for high grade lymphomas, constitutional symptoms (P<0.1), ECOG II, III, IV (P=0.0185) and also CNS involvement (P<0.1) for the intermediate group. There were no variables related to the survival for low-grade lymphomas.

**CONCLUSIONS:**

The intermediate grade lymphomas were more compatible with data found in the literature, probably because of the larger number of patients. In this specific case, the treatment did not have an influence on the survival.

## resumo

**CONTEXTO:**

Na doença de Hodgkin, cada estágio clínico ou patológico pode ser relacionado com a extensão da área envolvida e predizer a próxima região anatômica de risco para disseminação.

**OBJETIVO:**

Estabelecer os fatores prognósticos que melhor predizem sobrevida em LNH.

**TIPO DE ESTUDO:**

Estudo retrospectivo

**LOCAL:**

Disciplina de Hematologia e Hemoterapia, Universidade Federal de São Paulo - Escola Paulista de Medicina.

**PARTICIPANTES:**

142 pacientes com LNH diagnosticados entre fevereiro de 1988 e março de 1993.

**VARIÁVEIS ESTUDADAS:**

Tipo histológico, sexo, idade, raça, sintomas, sitação "performance", estágio, doença extranodal, desenvolvimento de Bulk, comprometimento mediastinal, envolvimento do SNC, infiltração da medúla óssea, nível de desidrogenose láctica, fenótipo imune.

**RESULTADOS:**

Ao primeiro estudo (113 pacientes), as seguintes variáveis tiveram uma pior influência na sobrevida: raça amarela (P<0.1); ECOG II, III e IV (P<0.1) e doença extranodal (P<0.1) para os linfomas de alto grau; sintomas constitucionais (P<0.1), ECOG II, III e IV (P<0.1) e envolvimento de SNC (P<0.1) para os linfomas de grau intermediário e o subtipo linfoplasmocitóide (P=0.0186) para os linfomas de baixo grau. Ao segundo estudo (93 pacientes), quando inclui-se o tratamento, as variáveis relacionadas a sobrevida foram : envolvimento de SNC (P<0.1) para o linfomas de alto grau; sintomas constitucionais (P<0.1), ECOG II, III, IV (P=0.0185) e envolvimento de SNC (P<0.1) para o grupo intermediário. Nenhuma variável relacionou-se com a sobrevida para os linfomas de baixo grau.

**CONCLUSÕES:**

Os linfomas de grau intermediário, provavelmente devido ao maior número de pacientes, foram mais compatíveis com os dados encontrados na literatura. Neste caso específico, o tratamento não influenciou a sobrevida.

## INTRODUCTION

In Hodgkin's disease, each clinical or pathologic stage can be related to the extent of the area involved and predicts the next anatomical region at risk for tumor dissemination. Thus, the Ann Arbor staging criteria^1,2^ are useful in planning treatment and determining the evolution of the disease. However, the biological behavior of non-Hodgkin lymphomas (NHL) is much more complex in relation to their presentation and natural history. In this way, discrepancies between NHL found in the various subgroups make it impossible to analyze them all together, and in particular we cannot predict survival based only on the anatomical areas involved.

Age, general condition at the time of diagnosis, serum lactate dehydrogenase (LDH) level, extranodal involvement and tumor burden assessment are the factors that have been shown to have prognostic value for survival in many studies.^3-7^ There are some variations between the different authors. We studied all these parameters in order to understand better which of these could predict survival among a set of patients of low socioeconomic level treated in a public hospital in the city of São Paulo.

## METHODS

An initial analysis of 142 patients with NHL was made, whose biopsies were reviewed and classified in accordance with the *Working Formulation* (WF)^8^ between February 1988 and March 1993 in our institution. From this, 29 patients were excluded due ineligible data, leaving the 113 patients that constituted our first study. A second analysis was carried out on 93 patients who completed their follow-up and could be evaluated as regards their treatment.

The staging of the disease were done before and after treatment. The tests performed included: complete and differential blood cell count; biochemistry test, particularly the LDH level; chest x-ray; chest, abdomen and pelvis CT scans; and bilateral bone marrow biopsies. Some special procedures such as lumbar puncture, brain CT or percutaneous liver biopsy were performed only if there was clinical suspicion of local involvement. Each patient was characterized using the following remission criteria after treatment: complete remission (CR), defined as a complete absence of any clinical or laboratory (biochemical or radiological) signs of disease maintained for at least four sequential weeks; partial remission (PR), defined as a reduction in tumor burden of 50% or more, measurable in two dimensions at the same time; refractory disease (RD), defined as a reduction in tumor burden of less than 50% compared with the original size, or a complete failure of response to treatment, or evidence of growing tumor burden during two sequential therapy cycles. The disease free-survival (DFS) was taken as the length of time between CR and the last consultation or the relapse. The overall survival (OS) used the same criteria for all patient situations.

The following clinical variables were evaluated: age in years; sex; race; general condition (performance status) in accordance with the Eastern Cooperative Oncology Group (ECOG);^8^ extranodal site involvement; presence of bulk disease defined as a tumor burden mass larger than 10 cm in one diameter or occupying more than one third of the mediastinum; and CNS involvement. The LDH level was scored at three levels: ≤ 225 U/dl; > 225 and < 450 U/dl; and ≥ 450 U/dl. Biopsies from different disease sites, mostly lymphoid nodes, were reviewed in the Pathology Department and reclassified according to the WF. When there were plenty of samples, immunohistological studies were done on the B (CD-20) and T (CD45RO) markers using the ABC method.^9^ The clinical stages were determined in accordance with the Ann Arbor classification. Seven different, non-randomized chemotherapy regimens were used. These were chosen based on the initial WF histological classification. The second and third generation schemes such as Macop-B,^7^ PromaceCYTAbom^8^ and the BFM-83 high-risk protocol^9^ were used for high grade groups. CHOP,^10^ BACOP^10^ and MVPP^11^ were used for intermediate groups. For low grade lymphoma, COP^11^ and MVPP^11^ were the most often used protocols.

*Statistical Methods.* Survival curves were plotted according to the method of Kaplan and Meier.^12^ Statistical significance among curves was determined by the Cox-Mantel method, measuring variables with two categories of results, and the generalized Wilcoxon test for those variables with more than two categories of results.

## RESULTS

Patients’ parameters in relation to the grade of lymphoma are shown in Tables 1 to 3 and the distribution of the 93 treated patients among the different chemotherapy schemes is shown in Table 4.

**Table 1:** Characteristics of patients with high grade lymphoma

Variables	1^st^ analysis		2^nd^ analysis	
	n°patients	*P-value*	n°patients	*P-value*
**Histological subset**				
Immunoblastic	12	0.42	09	0.54
SNCC	02		01	
Lymphoblastic	03		03	
Lymphoblastic	04		03	
**Sex**				
Female	05	0.06	11	0.15
Male	16		05	
**Age (years)**				
< 60	19	0.92	14	0.42
> 60	02		02	
**Race**				
White	15	0.04	11	0.07
Black	04		04	
Yellow	02		01	
**B symptoms**				
Present	13	0.15	09	0.62
Absent	08		07	
**Performance status**				
0	03	<0.01	02	0.52
1	12		10	
2	04		03	
3	04		01	
4	01		00	
**Stage**				
I	02	0.09	02	0.13
II	07		04	
III	06		05	
IV	06		05	
**Extranodal disease**				
Present	03	<0.01	03	0.06
Absent	18		13	
**Bulk disease**				
Present	10	0.63	08	0.48
Absent	11		08	
**Mediastinal disease**				
Present	06	0.63	04	0.87
Absent	15		12	
**CNS involvement**				
Present	01	0.14	01	<0.01
Absent	16		15	
**BM infiltration**				
Present	05	0.30	04	0.17
Absent	16		12	
**Level of DHL**				
≤ 225 U/dl	04	0.88	04	0.14
>225 and <450 U/dl	06		05	
≥ 450 U/dl	04		02	
**Immunophenotype**				
B	09	0.50	06	0.65
T	01		01	

SNCC = malignant lymphoma small non-cleaved cell; DSCC = malignant lymphoma diffuse small cleaved cell; DMSLC = malignant lymphoma diffuse mixed small and large cell; DLC = malignant lymphoma diffuse large cell; FMSCLC = malignant lymphoma follicular mixed small cleaved and large cell; BM = bone marrow.

**Table 2:** Characteristics of patients with intermediate grade lymphomas

Variables	1^st^ analysis		2^nd^ analysis	
	n°patients	*P-value*	n°patients	*P-value*
**Histological subset**				
DSCC	26	0.22	20	0.54
DMSLC	21		17	
DLC	18		15	
**Sex**				
Female	34	0.29	28	0.25
Male	31		24	
**Age (years)**				
< 60	47	0.96	41	0.13
> 60	18		11	
**Race**				
White	43	0.09	34	0.68
Black	22		18	
**B Symptoms**				
Present	43	>0.01	34	>0.01
Absent	22		18	
**Performance status**				
0	09	>0.01	06	0.02
1	38		33	
2	14		11	
3	02		02	
4	02			
**Stage**				
I	02	0.42	01	0.29
II	19		17	
III	16		12	
IV	28		22	
**Extranodal disease**				
Present	34	0.48	30	0.36
Absent	31		22	
**Bulk disease**				
Present	39	0.11	30	0.07
Absent	26		20	
**Mediastinal Disease**				
Present	20	0.87	18	0.65
Absent	45		34	
**CNS involvement**				
Present	04	<0.01	04	<0.01
Absent	39		39	
**BM Infiltration**				
Present	24	0.17	36	0.09
Absent	40		16	
**Level of DHL**				
≤l 250 U/dl	14	0.06	10	0.14
>250 and <450 U/dl	27		24	
≥ 450 U/dl	12		09	
**Immunophenotype**				
B	31	0.50	23	0.24
T	07		05	

NCC = malignant lymphoma small non-cleaved cell; SCC = malignant lymphoma diffuse small cleaved cell; DMSLC = malignant lymphoma diffuse mixed small and large cell; DLC = malignant lymphoma diffuse large cell; FMSCLC = malignant lymphoma follicular mixed small cleaved and large cell; BM = bone marrow

**Table 3:** Characteristics of patients with low grade lymphomas

Variables	1^st^ analysis		2^nd^ analysis	
	n°patients	*P-value*	n°patients	*P-value*
**Histological subtype**				
FMSCLC	12	0.02	11	0.29
Lymphoplasmocytoid	03		02	
Small Lymphocytes	12		12	
**Sex**				
Female	11	0.14	10	0.19
Male	16		15	
**Age (years)**				
< 60	24	0.98	23	0.45
> 60	03		02	
**Race**				
White	18	0.21	17	0.15
Black	09		08	
**B Symptoms**
Present	20	0.10	19	0.06
Absent	07		06	
**Performance Status**				
0	04	0.21	04	0.21
1	14		14	
2	07		05	
3	02		02	
4	00		00	
**Stage**				
I	03	0.26	03	0.26
II	03		03	
III	07		07	
IV	14		14	
**Extranodal disease**				
Present	07	0.62	05	0.48
Absent	20		20	
**Bulk disease**				
Present	04	0.65	04	0.7702
Absent	23		21	
**Mediastinal disease**				
Present	10	0.29	10	0.52
Absent	17		15	
**CNS involvement**				
Present	02	0.56	02	0.56
Absent	13		13	
**BM infiltration**				
Present	11	0.94	08	0.79
Absent	16	0.94	17	
**Level of DHL**				
≤l 250 U/dl	06	0.69	06	0.69
<250 and <450 U/dl	08		08	
≥ 450 U/dl	05		05	
**Immunophenotype**				
B	18	0.42	16	0.60
T	03		03	

NCC = malignant lymphoma small non-cleaved cell; DSCC = malignant lymphoma diffuse small cleaved cell; DMSLC = malignant lymphoma diffuse mixed small and large cell; DLC = malignant lymphoma diffuse large cell; FMSCLC = malignant lymphoma follicular mixed small cleaved and large cell; BM = bone marrow

**Table 4:** Distribution of 93 patients with non-hodgkin lymphomas according to the treatment received

	Lymphoma grade		
	High	Intermediate	Low
Macop-B	6	14	4
Promace-CytaBOM	1	7	3
BFM-83	1	3	3
MVPP	1	12	2
BACOP	2	13	8
CHOP	5	2	2
**Total**	**16**	**52**	**25**

Comparisons between types of treatment in each lymphoma grade. High P=0.7;Intermediate P=0.16; Low P=0.27

The remission rates for high-grade lymphomas were: 62% CR, 25% PR and 12% RD; for the intermediate group: 50% CR, 27% PR and 23% RD; and for the low grade group: 44% CR, 32% PR and 24% RD. MACOPB and CHOP were the treatments analyzed for high grade lymphoma and their CR were 66% and 40% respectively (P = 0.777). For the intermediate grade, the rate of CR to the protocols analyzed were: CHOP 61%, BACOP 41% and MACOP-B 50% (*P* = 0.1642). The median 5-year survival rates were: OS 62% and DFS 52% for high grade lymphoma, 57% and 49% for intermediate grade and 63% and 52% for low grade lymphoma respectively.

## DISCUSSION

We analyzed patients with NHL divided into three subgroups in accordance with the WF classification and evaluated the variables that could demonstrate prognostic value. A second statistical study was done to check the influence of treatment on the survival for each variable studied at the first step.

The intermediate grade lymphomas presented results comparable to those described in the literature, probably because this was the largest group of patients. In this group the variables associated with worse prognosis were: B symptoms (P< 0.01); performance status III and IV (P< 0.01) and CNS involvement (P = 0.006). There are reports that patients with a diagnosis of performance status of III and IV is related to high cell proliferation and this fact could have implications of an unfavorable prognosis.^14^ It is well known that better therapeutic results are associated with longer survival in patients that have a good performance status at the diagnosis.^5-7,13^

Involvement of the CNS generally implies frequently relapsing disease.^15^ Some studies have suggested that patients with bone marrow involvement have a higher risk of presenting CNS infiltration.^15^ This aspect was observed in our study.

The small number of patients with high and low grade lymphomas made it impossible to demonstrate statistically some important variables reported elsewhere such as: bone marrow and CNS involvement, bulk disease, mediastinal disease, high levels of LDH and age above 60 years.^15^

For high grade lymphomas the following variables were related to worse disease: yellow race (P = 0.003), ECOG III and IV (P < 0.01) and extranodal disease (P < 0.01). After including the treatment, the only variable associated with an unfavorable prognosis for survival was CNS infiltration (P < 0.01). Many reports have demonstrated that extranodal disease, especially if there are two or more involved sites, is associated with worse evolution.^3,16^ This is valid for all kinds of lymphomas. This fact, even if negated by others, makes us think that it may be necessary to have other factors associated with the extranodal disease in order to cause a worse prognosis.^5,6^ For low grade lymphomas, only the lymphoplasmocytoid subset (P = 0.018) had prognostic value, and in the second study no variable was associated with treatment and survival.

The overall survival and DFS for all grades of the WF classification are quite similar to those of other studies, although our patients were treated with different non-randomized protocols. Costa et al.^7^ had an OS of 54% and a 5-year DFS of 52% in a series of 54 high and intermediate grade lymphomas, most of which with diffuse histological patterns and advanced stage disease. Klimo & Connors^17^ found a median 8-year survival of 62% and a DFS of 52% in 126 patients with diffuse large cell lymphoma. For the low-grade lymphoma, the OS and DFS were comparable to the many other studies that included patients with advanced stage disease.^3^

A relationship between a high level of LDH and a worse survival for NHL has very often been reported.^5,14,18^ Schneider et al.^21^ pointed out the importance of this marker even when not considering the extent of the tumor nor the bulk mass burden. In our intermediate group, an LDH higher than 450U/dl had some relation with survival, with a p-value close to significance (P = 0.055).

Bone marrow involvement was not related to unfavorable survival in the three studied groups. We analyzed the presence or absence of focal or diffuse infiltration. For the intermediate grade lymphomas, there was a tendency towards diffuse marrow involvement and worse survival (P = 0.086). Data from the National Cancer Institute, Bethesda, USA, has postulated that marrow involvement means a poor prognostic factor for survival. A similar analysis performed at the M.D. Anderson Hospital was not able to predict this variable as an unfavorable factor. However, the association of bone marrow involvement, high LDH level and a high rate of cellular proliferation seems to have some prognostic meaning.^14^ In both of our statistical analyses immunological phenotyping did not influence the survival of the three different groups.

Our treatment was based on the first histological WF classification, but 43 of the 113 evaluated patients from the first study had their histological sub-type changed after review. Thus, the philosophy of basing treatment on the initial diagnosis generated a miscellany of analyses. Despite having a small sample size, especially when analyzing each chemotherapy scheme in isolation, our data are similar to those in other reports.^8,20^ Although MACOP-B seems to be better than CHOP for CR rates, we can conclude that they are similar when considering OS and DFS. This conclusion has been confirmed by other reports in the last five years.^21^

For the largest group, the intermediate lymphomas, there were no differences between CHOP, BACOP and MACOP-B relating to survival (P = 0.1642). When considered separately, CHOP showed a better rate of CR, 8/13 (61.50%), but this was not significant. At present, there is a world trend to use CHOP as the treatment schedule for aggressive lymphoma due to results that are similar and comparable to more toxic third-generation protocols.^7,8,13^

For low-grade lymphomas our results are slightly inferior to those of other reports, but are comparable to studies that included advanced stage disease.^22^ Even though 28% of those patients were treated with third-generation schemes and the others with CHOP, BACOP, MVPP or COP, no chemotherapy scheme was seen to be superior in relation to the prognosis (P = 0.2717). New treatment forms are being used for low-grade lymphomas, especially purine analogs, inter-feron and allogeneic and autologous bone marrow transplantation. However their efficacy is still limited when compared with classic treatments such as usage of alkylating agents or aggressive chemo-therapy.^23,24^ We believe that new studies and protocols are necessary in order to have a more precise conclusion about the best treatment for low grade lymphomas.^25^

A new attempt at classifying NHL has recently been made. In this new classification, lymphoproliferative disorders are divided into B, T or NK diseases.^26^ The cytogenetic characteristics and oncogenesis with their respective molecular rearrangements are considered in trying to describe each known pathology as an isolated entity. It is designated the REAL classification, signifying a consensus between American, European and Asian hemato-pathologists. This classification appears to have achieved the ideal, but more time and experience will be required before reaching a definitive conclusion on such a complex subject.

It is clear that we need to bring together clinical hematologists, pathologists and cytogeneticists in Brazil, in order attain a better understanding of the complex setting of this disease. Cooperation between institutions in different areas of the country could achieve clinical and therapeutic results. Only in this way will there be a commonality of spirit leading to a better understanding of NHL in Brazil.

## References

[1] Carbone PP, Kaplan HS, Musshoff K (1971). Report of the committee on Hodgkin's disease staging. Cancer Res.

[2] Rosenberg SA (1977). Validity of Ann Arbor staging of the non-Hodgkin's lymphomas. Cancer Treat Rep.

[3] Romaguera JE, McLaughlin P, North L (1991). Multivariate analysis of prognostic factors in stage IV follicular low-grade lymphoma: a risk model. J Clin Oncol.

[4] Velasquez WS, Jagannath S, Tucker SL (1989). Risk classification of the basis for clinical staging of diffuse large cell lymphoma derived from 10-year survival data. Blood.

[5] Vitolo U, Bertini M, Brusamolino E (1992). Macop-B treatment in diffuse large-cell lymphoma: identification of prognostic groups in an Italian multivariate study. J Clin Oncol.

[6] Shipp MA, Harrington DP, Klatt MM (1986). Identification of major prognostic subgroups of patients with large-cell lymphoma treated with m-Bacod or M-Bacod. Ann Intern Med.

[7] Costa AM, Froimtchuk MJ, Robinowits M, Olivetto LO, Allanse Gil RA (1994). Long-term results with Macop-B in the treatment of aggressive non-Hodgkin lymphomas. Am J Clin Oncol.

[8] (1992). Summary and Description of a Working Formulation for Clinical Usage: National Cancer Institute sponsored study of classification of nonHodgkin's lymphomas. Cancer.

[9] Pilleri AS, Sabatini E, Falini B (1991). Immunohistochemical detection of the multidrug transport protein P-170 in human normal tissues and malignant lymphomas. Histopathol.

[10] Longo D, DeVita VT, Duffey P (1987). Randomized trial of Promace-Mopp (PM) (day 1 and 8) vs. Promace-CytaBOM (PC) in stage II to IV aggressive non-Hodgkin's lymphoma. Proc Am Soc Clin Oncol.

[11] Hoelzer ET, Löffer H, Bodenstein H (1984). Intensified therapy in acute lymphoblastic and acute undifferentiated leukemia in adults. Blood.

[12] Yi PI, Coleman S, Saltz L (1990). Chemotherapy for large-cell lymphoma: a status update. Semin Oncol.

[13] Young RC, Longo DL, Gladstein E (1988). The treatment of indolent lymphomas: watchful waiting for aggressive combined modality treatment. Semin Hematol.

[14] Kaplan EL, Meyer P (1958). Non-parametric estimation from incomplete observation. J Am Stat Assoc.

[15] Silvestrini R, Costa A, Boracchi P, Giardini R, Rilke F (1993). Cell proliferation as a long term prognostic factor in diffuse large-cell lymphomas. Int J Cancer.

[16] Jagannath S, Velasquez SW, Tucker SL (1986). Tumor burden assessment and its implication for a prognostic model in advanced diffuse large-cell lymphoma. J Clin Oncol.

[17] Shipp AM, Yeap YB, Harrington DP, Klatt MM (1990). The m-Bacod combination chemotherapy regimen in large-cell lymphoma: analysis of the complete trial and comparison with the M-Bacod regimen. J Clin Oncol.

[18] Kirn D, Mauch P, Shaffer K, Pinkus G (1993). Large-cell and immunoblastic lymphoma of the mediastinum: prognostic features and treatment outcome in 57 patients. J Clin Oncol.

[19] Klimo P, Connors J (1985). MACOP-B chemotherapy for the treatment of diffuse large-cell lymphoma. Ann Intern Med.

[20] Danieu L, Wang G, Koziner B (1986). Predictive model for prognosis in advanced diffuse histiocytic lymphoma. Cancer Res.

[21] Schneider RF, Seibert K, Passe S (1980). Prognostic significance of serum lactate dehydrogenase in malignant lymphoma. Cancer.

[22] Miller TP, Lippman SM, Spier CM (1988). HLA-DR (Ia) immune phenotype predicts outcome for patients with diffuse large-cell lymphoma. J Clin Immunol.

[23] Gordon LE, Harrington D, Andersen R (1992). Comparison of a second generation combination chemotherapy regimen (m-Bacod) with a standard regimen (Chop) for advanced diffuse non-Hodgkin's lymphoma. N Engl J Med.

[24] Lyang R, Chiu E, Lake SL (1991). Management of low-grade lymphomas in Hong Kong Chinese. Oncology.

[25] McKelvey EM, Gottlieb JA, Wilson HE (1976). Hydroxydaunomycin (adriamycin) combination chemotherapy in malignant lymphoma. Cancer.

[26] Portlock CS, Rosenberg SA (1979). No initial therapy for stage III and IV non-Hodgkin lymphomas of favorable histologic types. Ann Intern Med.

[27] Cheson B (1993). New chemotherapeutic agents for the treatment of low grade non-Hodgkin's lymphomas. Semin Oncol.

[28] Chan JKC, MBBS, Banks PM, Cleary ML (1993). A revised European-American classification of lymphoid neoplasms proposed by the international lymphoma study group: a summary version. Am J Clin Pathol.

